# "The Hospital" Nursing Mirror

**Published:** 1899-11-11

**Authors:** 


"The Hospital" Nursing Mirror.

Being the Nursing Section of "The Hospital."

[Contributions for this Section of "The Hospital" should be addressed to the Editor, The Hospital, 28 & 29, Southampton Street, Strand, London, W.C., and should have the word "Nursing" plainly written in left-hand top corner of the envelope.]

### Notes on News from the Nursing World.

### THE "PRINCESS OF WALES" HOSPITAL SHIP.

It will be the enviable lot of Miss Spooner, of the Royal Free Hospital, to be one of the nurses of the sick and wounded on board the "Princess of Wales" Hospital ship. That the vessel is to be chartered at all is due entirely to the thoughtfulness of Her Royal Highness, who characteristically placed at the disposal of the British Red Cross Committee the balance of money which was collected by her branch of the society at the time of the Egyptian campaign of 1885, directing that it should be devoted to the equipment of a hospital ship for the conveyance of the sick and wounded back to the shores of their country. The committee of the society accordingly secured the "Midnight Sun" for the purpose, and as soon as the vessel is altered and refitted for the special object required she will be re-christened the "Princess of Wales." The Marchioness of Lansdowne, Viscountess Wolseley, Lady Wantage, Mrs. Wilton Phipps, the Hon. Sydney Holland, and Sir Donald Currie form the committee nominated by the Princess to carry out the necessary details, while the Central Committee of the Red Cross Society are represented by Sir John Furley and Major Macpherson. It is expected that the vessel will leave Southampton towards the end of the month. In addition to fitting it up Her Royal Highness intends to spend £1,000 in luxuries and comforts for the invalided soldiers. The whole nation has been touched by this gracious practical manifestation of the sympathy felt by the Princess for our suffering soldiers.

### THE WORK OF THE CENTRAL BRITISH RED CROSS COMMITTEE.

At Reading on Friday Lord Wantage stated that, with the Government vessels already chartered, and the "Princess of Wales," there are now sufficient transport ships provided, and a generous offer to supply a large steam-yacht has therefore been declined by the Central British Red Cross Committee. The work of the latter to the present date may be thus summed up. Colonel J. S. Young has proceeded as Red Cross Commissioner to South Africa amply provided with funds and material to act, as may appear best on the spot, in co-operation with the principal medical officer of the field force, with a view to aiding the sick and wounded, not only of our own troops, but also of the South African Republic and Orange Free State. Equipment for fitting up a hospital train, to be called the "Princess Christian," has been sent out to Durban; similar equipment is about to follow; and a complete hospital train of seven carriages, with kitchen and all necessary accessories, is being constructed at Birmingham, 400 men being at work on it night and day. Sir John Furley will be in charge of this train. Ample provision has been made by the Army Nursing Reserve to supply additional highly-trained nursing sisters to the various military hospitals. Forty have already taken over duties, and there remain 100 still on the list for employment as occasion arises. The St. John Ambulance Brigade is prepared to meet any requirements that may arise in connexion with the supply of ambulance officers and hospital orderlies, and several members of the brigade are now under orders to hold themselves in readiness. These, and the preparation in conjunction with the Special Committee of ships to be fitted at the expense of the Princess of Wales's branch of the National Aid Society, show how vigorously Lord Wantage and his colleagues have discharged and are still discharging their duties.

### NURSES AND THE WAR.

We are officially informed by the Secretary of State for War that the undermentioned nurses of the Army Nursing Service and the Army Nursing Service Reserve have either embarked or are under orders to embark for South Africa: A.N. Service—M. Russell, I. Hoadley, D. D. Tripp, C. K. E. Steel, S. Y. Snowdon, A. C. L. Anderson, A. Guthrie, I. A. G. Kinahan, E. Maclean, S. J. Brown, E. A. Cox, M. C. Rose, C. H. Potts, E. M. Todd, A. J. Sammut, M. G. Hill, M. R. Makepeace, L. B. A. Drury, A. A. Murphy, H. L. Neale, L. Hardement, M. L. De Montmorency, E. M. Beck, L. E. Steen, W. Potter, M. E. Harding, M. C. S. Knox, M. Mark, L. M. Todd, E. J. Martin, G. E. Saunder, A. E. Tait, A. Garriock, A. Nixon, A. R. Rose-Innes, R. T. Macintyre, A. Barker, L. M. Culverwell, M. Wilson, D. Palmer, M. C. F. K. Cole, C. H. Keer, and H. T. Young. A.N.S. Reserve—M. Lowe, P. Watson, E. M. Whiteman, and E. Laughton.

### PERSONAL NOTES ABOUT THE WAR NURSES.

The names of most of the war nurses have already been mentioned in these columns, but several additions will be observed in the list of the Secretary for State. We therefore print it in full for the convenience of our readers. Miss Jane Hoadley was trained at the Royal Infirmary, Edinburgh, and has been a sister in the Army Nursing Service since September, 1893. Miss Selina Isabella Snowdon was trained at the Cumberland Infirmary, Carlisle, and entered the Army Nursing Service in June, 1894. Miss Emma Martha Todd was trained at the Royal Free Hospital, Gray's Inn Road. Miss Mary Grenfell Hill was trained at Addenbrooke's Hospital and St. Mary's Hospital, subsequently acting as staff nurse at the West London Hospital until March, 1893, when she joined the Army Nursing Service. Miss Martha Mark was trained at the Jenny Lind Infirmary, Norwich. Miss Mary Cecil F. K. Cole has been attached to the Army Nursing Service since January, 1883, and received the Royal Red Cross in 1885.

### A GUY'S NURSE IN CHARGE OF HARRISMITH HOSPITAL.

Miss Margaret McKechnie, who is now head of the nursing staff at Harrismith, was trained at Guy's. The authorities of the Free State practically "commandeered" her, and though, being an Englishwoman, she naturally objected to have to work, as it were, for the other side, she accepted the position without protest, believing that the hospital would be in charge of Dr. Wilson, whom she knows well, and who is much liked. This was also the expectation of Dr. Wilson himself, or he would have left the town while he could; but, owing to the fact that he is a fine shot and a good rider, he has been ordered to fight against the English. Miss McKechnie is a native of Harrismith, where her mother and two brothers reside. One of the latter holds an office under the Free State Government, and will remain near his sister. The other has been sent with Dr. Wilson to the front. Miss McKechnie writes that her poor mother is almost broken-hearted that those amongst whom they have lived for years as friends should show so little consideration for them as to make one of her sons take arms against his own countrymen.

### THE AMERICAN HOSPITAL SHIP.

It has, after all, been decided that the nurses shall be under an American matron; and the five ladies will leave New York on Saturday for England. The four nurses selected were engaged in the Cuban War, and have, therefore, enjoyed valuable experience. They will be brought here free of charge by the Atlantic Transport Company. The whole *personnel* of the ship, except the chief medical officer, will consist of Americans. A committee of Americans resident in the French capital has been formed to join in the good work, and there will be a *café chantant* at Claridge's Hotel on Saturday, the 18th, on behalf of the fund. The "Maine" will leave Tilbury on Saturday, the 25th, and Lady Randolph Churchill will go to South Africa on the vessel as representing the Executive Committee.

### NOTABLE VOLUNTEERS.

Mrs. Richard Chamberlain, sister-in-law, and Miss Amy Chamberlain, niece, of the Colonial Secretary, who have gone out to South Africa to assist, as far as they can, in nursing the sick and wounded, intended originally to proceed to Vryburg, and, though not Romanists themselves, to join the nuns in that town. But it is now anticipated that they will go where their help is most urgently required. Mrs. and Miss Chamberlain have taken with them, in addition to medical appliances, quantities of wine and other luxuries not usually provided for the British soldier on active service. Many of these were gifts, and a firm of tobacco merchants sent the ladies 500 lbs. of tobacco.

### THE WAR AND THE LADIES OF THE NEEDLEWORK GUILD.

The Countess of Bradford, president of the Birmingham and Midland Counties Needlework Guild, desires through our columns to gratefully acknowledge the hospital suits made by the ladies of the Needlework Guild in response to her appeal of only a fortnight ago. The result, Lady Bradford says, has surpassed all expectations. "About 1,800 of these garments have been received, and they are still coming in: 1,000 are already on their way to Africa, shipped on the s.s. 'Pembroke Castle,' under the care of the Red Cross Society; 250 have been packed off to the Princess of Wales's hospital ship, and 200 more have been accepted by the American ladies for their hospital ship. The remainder will go out through the War Office direct to Durban; and of these 200 have already started, the rest follow at the end of this week." We agree with the president that "in coming so promptly to the front the Needlework Guild has shown what wonderful results can be attained by organisation and unity of purpose."

### IRISH NURSES AND THE SICK AND WOUNDED.

The Irish nurses are well to the fore in sending in contributions for the wives and families of Irish soldiers now on active service in South Africa. Mrs. Treacy, the superintendent, and the nurses of the City of Dublin nursing institutions have contributed £7 for the fund for the wives and children of the men of the Irish reserves, and £7 for the Irish Regiments Widows' and Orphan Fund, which, we are glad to observe, are both being generously supported in the sister island.

### THE PARCEL POST TO SOUTH AFRICA.

A correspondent desires to warn nurses who are sending out parcels by post to South Africa to take care not to accompany them by any kind of writing. She states that a friend had an album sent him from England, containing views, portraits, &c., with names written under them. "For each page so treated he was charged double letter postage, as well as a fine, so that before he could take possession of his present he had the pleasure of paying 25s.!"

### OUR CLOTHING DISTRIBUTION.

The lavish generosity with which the public have responded to the appeals for the benefit of the sick and wounded in the war encourages us to hope that the sufferers in the never-failing struggle against sickness and privation at home may fare equally well. An unobtrusive but substantial token of sympathy has hitherto been provided by the bundles of garments presented by our readers, which we have year by year had the privilege to collect and distribute. In 1898 no fewer than eleven parcels were either taken or sent to the various hospitals of the metropolis. What are we to send this year? The time is short, for they should arrive not later than December 19th, addressed to the Editor, and marked "Clothing Distribution." Anything in the shape of a useful garment for man, woman, or child is acceptable; and, although the weather at the moment is scarcely winterly, cold days are sure to come, and it would be pleasant to feel that at least a few of those whose strength has been reduced by illness will be able the better to withstand the variations of the English winter, owing to the kindly industry of members of the nursing profession.

### THE TRAINING OF FEE-PAYING NURSES AT BIRMINGHAM INFIRMARY.

At a meeting of the Birmingham Board of Guardians last week a recommendation of great importance by the Workhouse Infirmary Management Committee was carried unanimously. The result will be that the present plan of training nurses will be discontinued as soon as possible, and that in lieu thereof the training will be limited to probationers sent from the Workhouse Nursing Association only. The number is not to exceed ten at any period; the fee for training is to be £20 in each case for the first year, and for the second year such probationers are to continue to be trained without any further fee from them, or any salary paid to them by the guardians. It was remarked by Mr. Manton that the new arrangement would help smalle institutions as well as benefit the Birmingham Infirmary. Another guardian, Mr. Robinson, urged that the training of nurses should be undertaken by the Local Government Board. But there was great force in the reply of Mr. Manton that, although at various conferences he had heard arguments in favour of dealing with the question nationally by means of training schools, he "had never heard the people who argued on it come to any definite conclusion as to how it should be carried out."

### THE NURSES' HOME AT THE FLEMING MEMORIAL HOSPITAL.

Last week Mr. Watson-Armstrong opened the new home for nurses at the Fleming Memorial Hospital, Newcastle-upon-Tyne. It is nearly three years since the project of establishing it was first mooted, though the need had been felt much longer, but the committee of management determined not to build until the estimated amount of money required had been raised. Mr. Watson-Armstrong started the subscription list with £1,000, and in a very short period the total contributions came to upwards of £3,300. This was sufficient to justify the commencement of operations, and on the day the home was opened the sum of £1,051 was promised towards expenses and the cost of furnishing. The home is in three storeys, and affords accommodation for 20 bedrooms on the first and second floors. On the ground floor is a dining hall, common room, and waiting room, and altogether the premises and appointments are quite up to date. Mrs. Watson-Armstrong, in performing the opening ceremony, paid a high compliment to the matron and nurses. She said that she had made the round of the wards, and that throughout "there is the picture of cleanliness, comfort, and the utmost care." She added: "It is a pitiful sight to see the suffering children, but it is a cheerful and consoling sight to see them in the enjoyment of as much care as could possibly be enjoyed by such sufferers of any rank."

### ENTERTAINING THE LEEDS NURSES.

The entertainment by the Lady Mayoress of Leeds of a number of nurses at the Lord Mayor's private residence, Abbey House, Kirkstall, has been followed by another reception in the rooms of the Lord Mayor at Leeds Town Hall. On the latter occasion nearly a hundred trained nurses connected with the various institutions in the Town Hall were present. The amusements provided by the Lord and Lady Mayoress for their guests included a representation of the farce, "Poor Pillicoddy," which was admirably acted by nephews and nieces of the host and hostess.

### TRADE DISCOUNT FOR NURSES.

Our attention has been drawn by a nurse in the provinces to a circular issued by a London firm, in which it is stated that nurses will be supplied with their preparations at the same prices as the medical profession, "since we think it only fair that they should make a profit out of the dressing they obtain for their patients." Our correspondent suggests that this encourages waste and carelessness in dealing with the property of another in order to increase the number of dressings used, and asks our opinion on the subject. As a matter of practice nurses ought not to supply dressings at all; it is a question for the doctors. There is no objection to a firm offering a nurse the usual trade discount, but there is a strong objection to her acceptance of it. If a nurse recommends any special article for use it should be always because she has found it satisfactory, not because she has a pecuniary interest in it.

### UNTRAINED NURSES AT WORK IN IRELAND.

A correspondent sends us from Enniskillen a local paper containing a long account of an inquest on the body of Mrs. Ward, the wife of a Castleblayney hotel-keeper, who died from asphyxia, the result of narcotic poisoning. The feature of interest to nurses in the report is that the two persons engaged in nursing the deceased were untrained. Mrs. Mary Hughes deposed at the inquest that "she had had no professional experience of nursing, and that she told the husband of the deceased so before she was engaged." In the case of Mrs. Rice, her only experience of nursing, as set forth at the inquest, was that she had nursed a delicate child for about five months, and that two years ago she was in the Children's Hospital, Temple Street, Dublin, as a probationer for about six months. Asked why, in such circumstances, she wore a uniform, Mrs. Rice replied, "I suppose there was a certain amount of pride attached to the wearing of it." She denied, however, that she led Mr. Ward to believe that she was a qualified nurse. It is fair to state that no blame for the result of the case was attached by the jury to the nurses, but the Coroner said, "It was quite evident that Nurse Rice had been giving deceased medicine unknown to Dr. Wilson." A trained nurse would not think of doing such a thing.

### MEMORIAL TO NURSES IN AMERICA.

The Spanish-American War nurses have asked permission of the Secretary of War to erect a monument in the national cemetery at Arlington to the trained female nurses already buried in the cemetery, and also to those who may hereafter die in the service of their country. The monument is to cost not less than 10,000 dollars, and the design is to be submitted for approval to the Secretary of War. In the unexpected event of his refusal to sanction the scheme, it is in contemplation to place the Memorial in Riverside Park, near New York.

### SHORT ITEMS.

The friends and supporters of the Women's Convalescent Home Association have agreed to raise a special fund for the purpose of providing free beds for the wives and daughters of reservists on active service. At present two beds have been specially reserved, allowing for 40 or 50 cases, but more will be provided if required.—At a meeting in aid of the Leamington District Nursing Association the Hon. A. Lyttleton spoke strongly in its favour, and said: "Hospital and nursing establishments have not yet come within the range of municipalisation, and he was not sure that they were any the worse for that."—At the quarterly meeting of the Scotland Council of Queen Victoria's Jubilee Institute for Nurses it was stated that the names of 21 nurses when training was completed during the preceding six months had been recommended for the Queen's roll, and that new affiliated branches had been formed at Forth, Wishaw, and Armadale.—At the quarterly election of probationers for training in the hospitals connected with the City of Dublin Nursing Institution 22 out of the 48 candidates who presented themselves were successful.—It has been decided by the Hereford Board of Guardians to increase the nursing staff by the appointment of two additional assistant nurses.—Miss M. B. Ireland has declined to take up the appointment of nurse under the Bridgend Board of Guardians in consequence of a new stipulation that she was to do night and day duty.—The first Dutch Red Cross ambulance for the succour of the Boer sick and wounded arrived at Naples on Tuesday, in charge of Professor Konteveg. The party included seven nursing sisters.

### Lectures to Surgical Nurses.

By H. A. LATIMER, M.D. (Dunelm), M.R.C.S. of Eng., L.S.A. of London, Consulting Surgeon, Swansea Hospital; Past President of the Swansea Medical Society; Lecturer and Examiner of the St. John Ambulance Association, &c.

RESULTS OF ANEURISMS.—METHODS OF CURE.

AT the conclusion of my last lecture I said that I should give you one or more illustrations of the injurious effects produced by aneurisms. Those I have already adduced concerned arteries lying inside cavities of the body, and were therefore ones which mostly interested physicians, though it is true that the modern surgeon, from whom hardly anything is sacred, has attempted to cure disease here, as elsewhere, by operative interference. At present I will deal with such aneurisms as come legitimately within the surgeon's domain. For this purpose I cannot take a better example than that afforded by aneurism of the popliteal artery—the main vessel of the leg as it passes down the back of that limb, behind the knee joint. This is a favourite situation for this disease to manifest itself, largely because the vessel is subjected to much strain at this spot.

When affected by aneurism the artery will enlarge and will have the expanding impulse I have already spoken of, and very soon weakness and pain will occur. If the disease progresses very serious results now impend, for gangrene of the whole limb may be caused by interferencewith blood supply, or by the escape of some fibrin from within the walls of the artery; or the artery itself may rupture at the point of aneurism, and a very serious and sudden hæmorrhage may take place. These results of the disease are common to all aneurisms, and show how necessary it is to undertake their cure whenever that is possible. In rare cases the cure may occur spontaneously, but this happy result is so seldom to be met with that it is quite a phenomenal event. It is, however, by imitating Nature that we arrive at a method of cure, and I will now proceed to tell you what that method is. I suppose all of you are familiar with the fact that blood is possessed of the property of "clotting." You have noticed that very shortly after blood has escaped from a vessel—say, for instance, from that one concerned in a bleeding nose—it separates, as it lies in a basin or other receptacle, into a fluid and a soft mass. This mass—the clot—is composed of a material called "fibrin," which has entangled in its meshes many of the cells which have been floating in the blood. It is by means of this fibrin that cut vessels become sealed, and the escape of blood from them is arrested; for, although very soft in the first instance, it becomes hard and dense later on, when it is said to have become "organised," in which state it has become a living part of the vessel, where it at first lay as a kind of semi-fluid glue.

Now it is by securing the services of this fibrin within the walls of the diseased artery that the circulation within it is arrested and the aneurism is cured. It was noted long ago by observers that if the inlet or outlet of the affected part of the vessel became blocked up by a clot of blood which had fortuitously lodged there fibrin would form, and, if all went well, the disease would be cured by the clot organising itself and so sealing up the artery. The same event would take place, it was found, if the circulation of blood through the artery was arrested by tying it in certain chosen positions, or by pressing upon it at a point before the blood could reach the aneurism. So the surgical treatment of this disease consists in applying one of these methods, and where the vessel is situated at a spot where the circulation of blood through the diseased area is controllable, the results of modern treatment are very good. Where it has been decided to apply the ligature we are helped in a remarkable manner in obtaining success by the fact that modern antiseptic surgery enables us to bury it in the limb without fear of its causing mischief. I consider that this matter of applying ligatures and sutures to deep-seated parts and leaving them there with a knowledge that no ill result will follow such a course is one of the greatest advantages which have accrued to us by the adoption of the antiseptic system introduced by Lord Lister. Before this great revolution in surgical methods was adopted it was thought to be necessary to leave the ends of a ligature hanging out of a wound, and the ligature was removed when it had separated from the vessel it had enclosed, by the primitive method of gently pulling it away. So two things were involved in this old way of tying vessels—the wound made in getting at the artery had to be kept open to allow of the subsequent removal of the ligature, and this, again, secured a septic track for the entrance of germs of disease, with all the liability to suppurative fever which followed such a contamination of the tissues. Contrast this old method with what now obtains! To-day the vessel is cut down upon and is tied, at a selected spot, with an antiseptic ligature, the ends of this ligature are cut short close to the artery, the wound is then closed by sutures, and, if all goes well, primary union is effected, and the ligature is never heard of again.

Sometimes the circulation through the aneurism is controlled by digital pressure, that is, by the thumbs of relays of students pressed upon the artery above the point where it is diseased; sometimes by a tourniquet; sometimes by bandaging a limb with an Esmarch elastic bandage. In internal arteries, out of reach of such methods, cure is sometimes effected by a semi-starvation diet, and by absolute rest and quietude in bed. The methods here, although differing greatly from each other, all effect a cure by a like result; they aim at allowing only a very languid flow of blood through the aneurism, so that clot may form there, and, having formed, may organise itself without fear of being washed away, and so obliterate the artery.

You may ask me how it is possible to block up the great main artery of a limb in this way without endangering the parts supplied by it with blood and so causing their mortification. It is certain that unless a part can receive a sufficiency of blood for its nourishment it will die, and so it is but reasonable to inquire how gangrene is escaped in these cases. The answer is given us by a knowledge of anatomy. Although the great blood supply of a limb in health is derived by one or more main arteries in it, communications between branches of this vessel and similar ones derived from other large arteries situated at a distance, exist. These branches may be very small in the first instance, but there they are all the same; and when the necessity arises for their use they enlarge for the direct purpose of taking up the burden of work which is interrupted by a closure of the main artery which has hitherto carried on the circulation in the limb. They then establish what is known as a "collateral" circulation, the word "collateral" being defined in the dictionary as meaning "On the side of; subordinately connected; indirect." To illustrate this point imagine a water-course which it has been decided to block for a short distance for some purpose or other. At a point above the blocking place a side channel is made, which, after running alongside the main course which is now blocked for a distance, is conducted afresh into the channel at a point below where the water has been prevented from flowing. Now you see that the current of circulation has been diverted, and that it is resumed below the blocked-up spot. Apply this to a limb, only multiply your diverting channels. You will then easily see that, if all goes well, an artery may be blocked by ligature, and that its current of circulation may be resumed below the ligatured part by means of the small channels which always exist as a part of the plan of circulation of blood in our bodies; and if the place of ligature is well chosen, with an anatomical knowledge of the collateral circulation of an artery, and the limb which is operated upon is placed in such a position as to be shielded from injury, and to be favourably helped in its circulation during such time as the burden and stress of affairs is being laid on the new channels, clot forms in the blocked part. This clot converts the hitherto pervious artery into a solid cord, and the aneurism is cured by complete obliteration of the vessel down to its point of re-entrance of blood from a side stream.

### The Colonial Nursing Association.

### II.—ITS PRESENT POSITION.

From the moment the Colonial Office accorded official recognition to the movement originated by Mrs. Piggott the valuable support afforded at the instance of Mr. Chamberlain encouraged her to renewed efforts on behalf of the Colonial Nursing Association. The supply of the nurses to Mauritius, which preceded the first appearance in public of the association, was followed in the spring of 1897 by the despatch of a nurse to Cyprus, where a local subscription fund had been raised. As in the case of Mauritius, the association provided passage, and guaranteed any annual deficit in the amount required for the maintenance of the nurse. It has, however, been from the outset the policy of the association to urge upon the colonies to which nurses are sent the duty of finding the wherewithal, if possible; and it is due to the people of Mauritius to state that they have not only refrained from calling upon the association to make up a deficit, but have also repaid the passage money of the two nurses sent out. In addition to the Crown colonies, the British communities in foreign towns are included in the scheme of the association, and among these Bangkok and Yokohama were the earliest to take advantage of it. A remarkable proof of the needs of Cyprus, where Government funds are only available for one nurse, was afforded by the fact that the nurse was called to her first case straight from on board ship, and went to a lady dangerously ill the moment she was landed.

The action of the Colonial Office in recommending the scheme to the support of the Crown colonies, had the immediate result of drawing fresh attention to the nursing question in our colonies, and in a short time several of them, in which up to that period no provision had been made, voted sums from Government funds for the purpose. The Gold Coast was the first to recognise the value of the association by allotting sufficient money for the establishment and maintenance of three nurses from England at the Government hospital at Accra. Thus was carried into effect the further object of the association to supply matrons and nurses in the Government hospitals, and the way paved for the installation in the hospitals of an English nurse, or nurses, available for employment for private patients. It was therefore at the request of the Colonial Office that in December, 1896, the Colonial Nursing Association selected two highly-trained nurses to proceed to Accra. They sailed in January, 1897, and a third in April. The colony of Lagos, which had previously possessed two English nurses in the Government Hospital, next applied to the association to send out a third. Meanwhile, St. Vincent and Dominica had asked for midwives, and Sierra Leone for two nurses.

The next to apply was Bermuda, and several of the Protected States of the Malay Peninsula; at that time the residents in these places were suffering severely from lack of trained nursing. Accompanying an application from Selangor for a nurse for private work was a touching letter from Mrs. Treacher, the wife of the British resident, stating that at the date of writing two English ladies were at death's door. One of these she described as having had to go through the most trying time of a woman's life without any trained aid whatever. In addition, she had lost her child; while the other lady had been obliged to go into the hospital for her confinement in order to secure the services of "the only nurse in the State," an Eurasian, and not, of course, a fully trained person. In Perak the association has two nurses and one Government matron at work.

So the work proceeded, and applications continued to steadily come in, Ceylon, Hong Kong, and Trinidad being amongst the colonies to whom nurses were despatched by the association in 1897. Matrons were supplied to Government hospitals at Cyprus and Trinidad. Here, it may be mentioned, that Mrs. Piggott, in urging Sir Edward Wingfield to make the association the recognised official channel through whom the Government get all their nurses for the Crown colonies, had not an extremely difficult task. Before the association was formed the Colonial Office used to send out the nurses. Their knowledge of nursing qualifications must necessarily be restricted, and the force of the argument that a body of ladies, some of them enjoying the advantages of nursing experience, must be better able to exercise sound judgment, was irresistible. To have left the Colonial Office channel open would also have deprived the association of the certainty of securing the best nurses. The arrangement that every nurse sent out to a Government hospital must go under the auspices, and by the direction of, the association has, while materially strengthening its position, obviously relieved the Colonial Office of duties which were neither congenial nor desirable. At the present moment one of the obligations which Mrs. Piggott undertakes is to personally correspond with every nurse who goes out on the private staff or to a post in a Government hospital in the colonies; while candidates who have successfully and suitably filled in a form of application are personally interviewed from time to time by the nursing committee and their references investigated. When a vacancy occurs, the committee again bring together a certain number of suitable candidates for selection, requesting at this interview the co-operation of any residents in the colony where the vacancy occurs who may happen to be in England at the time, thus securing advice and information as to the conditions of life in, and climate of, the place to which the nurse is to be sent. The new method of selection does indeed involve a good deal more labour than the old, but its overwhelming superiority cannot be challenged. All nurses are medically examined before appointment as to their fitness for tropical work.

The development of the association has been rapid. Demands from fresh quarters are constantly being made upon its resources. Ceylon, Bahamas, the Niger Protectorate, and East Griqualand are among the colonies which have invited its aid during the present year. Since the society was formed forty-two nurses have crossed the seas as its representatives, either to take up appointments as matrons or nurses in Government hospitals, or as private nurses. The founder has been honorary secretary from the start, and for three years she worked single-handed; but in July, 1898, it became absolutely requisite to appoint an assistant secretary. In addition to the council, which is composed of some of the most distinguished people, there is an executive committee, which also consists of well-known ladies and gentlemen, and is divided, for purposes of celerity, into sub-committees. The honorary officials of the association are: Auditors, Messrs. Pim, Waterhouse, and Co.; treasurer, Mr. W. A. B. Musgrave; solicitor, Mr. Fred. Dalton; and secretary, Mrs. Francis Piggott. The council meet annually, and the sub-committees as frequently as circumstances need. No colony applying for a nurse has to wait, and the Colonial Office can depend upon a vacancy being suitably filled at the shortest notice, for no less than 150 nurses, who have all been interviewed and found to fulfil the conditions of service, are willing to take up engagements under the Colonial Nursing Association whenever they can get them.

### Across the Seas.

### NIGHT DUTY IN AN INDIAN HOSPITAL.

By One of the Staff.

As a general rule we are limited to one night nurse for our hospital, which includes the main building, consisting of eight wards and four private rooms (the total number of beds being ninety-nine); two D.T. wards, and an obstetric ward. These latter are built at short distances from the body of the hospital, but in the same grounds. The eye ward, two surgical wards, and one medical—all for native male patients—are on the ground floor. The four private rooms, two at each end of the building; a native women's surgical and medical ward; two similar ones for European men and women respectively; and a Government one for native police are on the upper verandah, three flights of stone steps leading up to it from the centre hall.

The night nurse's duties are from a quarter past eight p.m. to a quarter past seven a.m. Each staff nurse has a night report book, in which she enters the orders for the night, and this is handed to the night nurse as she relieves the nurses of the several wards. The latter, in her turn, enters her night report in these books, and leaves them on the ward tables of the different wards at her last visit. The staff nurse and senior probationer only are relieved by the night nurse, the latter informing the junior pros. that they are relieved.

Besides the night nurse there are two students on general night duty (a different couple each night); one student on poisoning duty, who is changed every week; one ward servant and one sweeper—the two latter carrying stretchers, bringing hot water, and helping in any way needed with patients who are brought in during the night.

All these students and servants are allowed to sleep, and are only called up if necessary, as we have not a separate staff for the night, and there is the day's work before them.

About nine p.m. the ramoosie (night watchman) takes his lantern, to bring over the house surgeon. The night nurse meanwhile reads her reports through, and finds out the new patients, the day's operations, &c., to report to him as they go their rounds.

The hospital is none too well lighted, so that it is quite an imposing procession, the lantern being carried in front, followed by the house surgeon and nurse, then the students, and last of all the muccadum (the head servant responsible for the others being at their posts, &c.) The senior student brings his night-round book with him, in which any orders the house surgeon gives are entered. The ward servants and sweepers have all to be present in their respective wards except the two on night duty. On this occasion all the wards are visited, and night draughts, medicines, stimulants, &c., ordered, for as there is only one house surgeon he is not called up unless absolutely necessary; but no European case is admitted unless previously seen by him.

The ward servants of the wards for which medicines, &c., have been ordered take bottles, &c., to the dispensary, putting them, when compounded, on their ward table, the night nurse, as each ward is visited, telling them how many are needed. Then the regular work of the night begins, usually in the form of ten o'clock feeds, or ipecacuanha treatment for liver or dysentery cases; so that long before you are ready the freshly ordered night draughts are waiting. In answer to reproachful looks I have often had to explain that I was the only nurse on duty.

The servants, not on duty, sleep in the front or back verandahs of their wards. As a general rule there are two or more patients in the back verandah of each ward, cases which, though we had our full number of patients, were too serious to be refused, bringing up the total to considerably over a hundred.

The night work consists principally of feeds, temperatures, stimulants, medicines, ice-bags, and poultices, occasionally nutrient enemas, and the usual constant watch for hæmorrhage. We have a great deal of typhoid, so that with three, sometimes six, of these, and five or more major operations, besides the other cases, the night work is most trying. I have often felt so bewildered that my head has been in a perfect whirl, and I have not known which thing to begin with; frequently before a round of feeds, for instance, is finished, medicines are overdue.

Then there are visits to be paid to the obstetric ward, and as a rule there is at least one little scrap of humanity for whom you musc heat dill water and administer it, besides a drink of milk for some mother, and one has to see generally that all is well. When a case for this ward is admitted during the night, the nurse in charge of the ward is called up, and she will decide after seeing the patient whether her probationer shall be summoned or not. The nurses' home is about three minutes' walk, on the other side of the main building from that on which the obstetric ward is built. There are also the D. T. wards to visit, so that when visiting any of these outer wards, or going to the home for the obstetric nurse, the night nurse cannot possibly know of anything happening in the hospital proper, and the result is that with the over anxiety one's nerves get badly out of order.

Occasionally a case is brought in which needs instant operation; the house surgeon is then sent for, and the patient is taken to the theatre. If there is no probationer on night duty, and the operation is likely to last some two hours, or is an extremely serious one, the day staff nurse of the ward to which it will belong is sent for, or a probationer to take over the night duty, but such a necessity is very rare.

It is very seldom, indeed, that a night passes without a case of some sort coming in, either an assault, accident, burn, alcohol, or opium poisoning, or a case for the obstetric, but these we usually manage to see to ourselves if there is a fairly capable student on duty; only when an opium case is too serious even to allow of the student attempting to pass the stomach-pump, or a fracture comes in, or tetanus, then the house surgeon is sent for. The senior student is supposed to send the "ramoosie" to tell the night nurse of any case which comes in, but the usual thing is for the night nurse to hear the cart rumbling in, and wake the "ramoosie" to call up the students, for I have myself never known a "ramoosie" keep awake all night long. Dead bodies are brought in by the police at all hours of the day and night. The night nurse helps with all the cases which come in during the night if she can possibly spare the time; moreover, the nurse often knows a very great deal more of practical work than the student.

The rule is for the night nurse to go all round her wards every two hours; the midnight and four a.m. rounds are heaviest. At these hours temperatures, medicines, poultices, feeds, &c., are entered in the night report books for two or three cases in each ward, so that for the twelve p.m. work it is necessary to begin at eleven o'clock in order to be finished by one a.m. It is this terrible rush which tells on one so much, but one gets through it in the end somehow, and seven a.m. at last strikes. At six a.m. the nurse begins to write up her night report books, entering new patients, fresh symptoms developed in old ones, &c. The making of beds, giving breakfasts round, &c., which falls to the night nurse in many of the home hospitals, is of necessity here left to the day nurse. No report is to be given or received *viva voce*, the recipient being severely reproved and having to bear the onus of any mistake which may arise.

After the arrival of the day nurses, the night nurse goes to the sister-in-charge in her office, at the home, and reports on all the serious cases, new admissions, &c. The rule is that she is to be in her room by 9 a.m., and not leave it again till 4 p.m. The term of night duty varies; I have done it for nearly six months at a time; at others for two, and so on. It is difficult to obtain a permanent nurse, as night work is especially trying in India.

Serious cases, as officers with typhoid, in our private rooms often have both day and night "specials," so that then the night nurse on general duty is relieved from that case. Very occasionally in the general ward the relations of two or more very serious cases may agree to pay for a "special" night nurse between them.

When the upper floor is very heavy a pro. is sometimes given for the lower floor, but it depends very much on the day cases and whether any of the nurses are out at private cases. The D.T. wards do not, I am glad to say, often hold D.T. cases, but such as gangrene, before operation, tetanus, mumps, puerperal fever, &c.; the wards being small are easily disinfected and white-washed; also any native official whose rank and way of life would make it equally unfitting to put him in the general native ward, or anywhere else. They are used for both medical and surgical wards, as needed; I once heard a medical officer say that they were the most useful wards in the whole hospital.

Our D.T. wards are by no means the padded-rooms which would present themselves to the imagination of many; they are built of stone and white-washed inside, with mud floors, —the windows having bars—so that disinfecting is soon accomplished. When a veritable D.T. case is admitted all the furniture is removed, and only a straw mattress put down on the floor.

The private rooms are furnished like ordinary bedrooms. For the use of these a fixed sum has to be paid, and so much per cent. in addition on the salary or income of the occupant; "special" nurses being charged for separately if engaged, either by the patient's friends, or on the recommendation of the medical officer in charge of the case. These rooms are quite cut off from the general ward, so that it is impossible for the nurse on general duty to give a serious case the necessary amount of attention. The rooms are very popular, and cases have to be refused sometimes unless they are willing to be screened off in the general ward for the time being.

From half-past five a.m. the night nurse who is wise keeps away from the back premises and the native wards as much as possible, for the first duty of a native's day is to "wash his mouth." It consists in repeatedly thrusting his fingers at least halfway down his throat, the noise being in the highest degree disgusting and upsetting after a night watch.

Pariah dogs infest the compound during the night, much to the disturbance of sensitive patients, and the bark is so peculiar that even those half a mile away are quite as irritating. The night nurse, when able, makes a round of the compound with the "Ramoosie" to try and clear some of them away. Inside the hospital is infested with cats, by no means tame, and chairs or some small article of furniture must be put up against the lockers of those patients on feeds, for although closed the cats undo the clasps very cleverly with their paws.

In the surgical wards the night nurse must watch those with splints on—Liston's, MacIntyre's, &c.—night being the favourite time to remove them. The splint will be in the bed safely enough, but quite unattached to the limb. This is especially the case with MacIntyres.

One morning my pro. came to tell me that a child in a Liston's had been taken away by his father between her five and six a.m. visits; the splints, bandages, &c., had all been taken off and left behind. We found out that the previous day the father had asked permission to take the child home, but had been told to wait for a few days. This is a rare thing, though. Patients in plaster of Paris are a constant source of anxiety to the night nurse, as the chances are greatly in favour of her finding him busily picking it off. I once had a villager in—an amputation of thigh, middle third—for mycetoma. On the very first night he took off every particle of dressing and bandage from the stump three times, just covering it with the antiseptic blanket. He made a splendid recovery; he was not off his head at all, but was quite uneducated and untrained, so he said that he had let the doctor sahib amputate his leg, but it would heal quicker if uncovered. These cases are a great anxiety. In my account of night duty I must not forget the "racoons," which infest the roof of the hospital, and sally down at night for food; they are very daring. Their footfall is very heavy, like a man with thick boots on. They are something like a cat, but have long snouts, with which they burrow. My readers will see that it is not only patients with which the night nurse, at least in some parts of India, has to contend.

### Novelties for Nurses.

### UNBREAKABLE WARE.

The "Thetford" unbreakable pulp ware is intended to supersede the all-too-destructible china ware in domestic use. With this end in view Messrs. W. B. Fordham and Sons, of York Road, King's Cross, have manufactured an immense variety of articles for the table, toilet, and household. They also have a special selection of articles for institutions. The material is strong, and will stand the usual cleansing processes and the action of hot water. The patterns are neat, and the general appearance closely resembles that of earthen and china ware.

### THE RONUK TRAVELLING CASE.

This travelling case consists of a neat little box containing an excellent polish for black kid boots, brown leather boots, and for bicycles or furniture, with a piece of sylvet polishing cloth with which to apply the polishes. With "Ronuk" as a polishing paste for furniture we were well acquainted, and have found it most excellent. The boot polishes, which are new to us, are most satisfactory. The little case would form a useful an inexpensive present to a nurse going on her travels. The address of Ronuk (Limited) is 83, Upper Thames Street, E.C.; but any grocer will supply the goods if ordered should they not have them in stock.

### Hospital Reform.

Lecturing on the subject of "Hospital Reform" at St. Martin's Town Hall on Tuesday night, Miss Honnor Morten, of the London School Board, strongly advocated public control of all hospitals. The system of voluntary hospitals, she maintained, was breaking down. A great disadvantage was that there were no women on the management. It was a matter which ought not to be dealt with by busybodies and philanthropists, whose main object was to get a peerage. Admitting that charitable hospitals were made bright and cheerful, and that friends were freely admitted, and that municipal or State hospitals might be dull and dreary looking, Miss Morten held that medical men got placed on hospital staffs for their own advancement. Extravagance and inefficiency, lack of uniformity, cooking of the accounts in order to show favourable statistics, were further defects. Objection to receiving moribund patients, and the passing them on from one hospital to another, formed a contrast to the system in Paris, where the ambulance plan for conveyance of patients to hospital was as perfect as the practice of our fire brigades.

### Death in Our Ranks.

### TYPHOID FEVER AGAIN.

We regret to hear of the death, from typhoid fever, of Nurse Norman, a probationer of the Royal South Hants Infirmary, Southampton. The sad event occurred last Friday, and the matron of the infirmary, writing us in respect to it, says: "Miss Norman was only twenty-two years of age. She was a charming girl, universally liked, most conscientious, and devoted to her work. Indeed, she was one of our most promising probationers."

### Echoes from the Outside World.

### AN OPEN LETTER TO A HOSPITAL NURSE.

There is very little official news from the seat of war just now, because both the railway lines and the telegraph wires running to Ladysmith have been rendered useless by the Boers, and we have to depend for information upon the pigeon post and upon reports given by natives, more or less trustworthy. Captain Kennedy is on his way out to South Africa with the Marconi wireless telegraphy apparatus, and when that is in working order—which will take four days after landing—the enemy will be powerless any longer to deprive us of news. The War Office seems to think that Sir George White can hold his own, and keep the Boers out of Ladysmith till reinforcements arrive. Apparently ten days must elapse before troops in a sufficiently large number to strike a decisive blow will be on the scene of action. It is sad to read that "in Thursday's action near Ladysmith the enemy were cut to pieces by the Lancers, Hussars, and Dragoons, helped by the infantry's bayonets. The enemy had displayed the white flag, and then, as usual, treacherously fired a volley on the troops as they advanced. This infuriated our men, who charged, cutting up the enemy without mercy, for which they howled," but one can hardly wonder. Colenso has been evacuated because we had not enough men to hold it, the troops have fallen back on Estcourt, and the enemy are closing in on Kimberley. Martial law has been proclaimed in the northern districts of Cape Colony, and it is hoped that this will have a good effect upon the Natal Dutch. Major Prince Christian Victor, son of Princess Christian, left Cape Town on Friday for Durban, and as he is going to Ladysmith to take up regimental duty there, one member of our Royal Family, in addition to so many scions of the nobility, will soon be in the thick of the fight. As to home news, the Queen—who, at Windsor on Saturday, is going to inspect a regiment of the Household Cavalry ordered to proceed to South Africa—has given £1,000 and the Prince of Wales £262 10s. to the Transvaal War Fund at the Mansion House, which has now reached a total of nearly £150,000. Considering the numerous demands upon the private purses of the Sovereign and the Heir Apparent, of which the public know nothing, these donations are most generous.

I have had another letter by this mail from my correspondent in Natal, who promises to send me information as often as she can. She has been staying at a country place a few hours' journey from Durban, situated close to the line of rail, and hearing that some of the volunteers who had been ordered out were passing to the front, she and three other girls walked through the drenching rain to Gillitts Station to bid their friends good-bye. She says: "We started off at two p.m., and marched along the line, arriving at the station like 'drowned' rats. The station master's wife proved to be an angel in disguise, for she dried our garments, gave us tea and cake, and quite entered into our enthusiasm, so much so that when she found we knew four or five of the officers and a good many of the privates, she offered, if we liked, to provide tea for them. You can imagine how we jumped at the offer, and very soon we had teapots, cups and saucers, and cake set out in the ladies' waiting-room. The first contingent of the Naval Volunteers and Durban Light Infantry came up at four, but our friends were not amongst them, and we had to wait till the Kaffir mail had passed before the others appeared. As soon as we spied those we knew we rushed up and invited them to our impromptu tea party. Some brought their chums with them, so that in a few moments we were helping at least fifty men to tea and cake, for which they seemed very grateful. It was dreadful not being able to give to all. But what could we do? We had only ten cups, so that our entertainment was naturally of a limited description. The men were in open cattle trucks. It was bitterly cold and raining fast. Already some of the younger boys' hands were frozen; but they would have to remain exposed to the elements till five o'clock next morning, when they would reach their destination. The officers at first were allowed carriages, but later on had to join the men in the trucks. They told us that the orders for them to go to the front were only given the day before, so that they had scanty time for preparation. As the men left Gillitts Station they gave us three cheers, and went away singing 'For they are Jolly Good Fellows,' looking so fit and well. And some we may never see again! The next day we learnt that the Durban Mounted Rifles were to pass through Gillitts Station. So in a thick, damp mist, with two natives as escort and a couple of lanterns, we again walked the three miles at half-past nine p.m. to wish them "God speed." We found the station coolie asleep, but as we were determined to make the little station look as bright as possible, we lighted every lamp in the place ourselves, and sat down to wait in company with three British workmen who, like ourselves, were full of enthusiasm. When our reward came it was only fleeting, for the three trains came quickly by; but in one the kindly engine-driver slowed down, and the men were able to lean out and cheer. Since then the 'Malta' troops, and the first detachment from India have gone up, and we have seen truck-loads of poor women and children refugees come by, riding in the open in the pouring rain."

My correspondent continues: "A young fellow who called in here on his way back from Maritzburg, where he went to join the Imperial Light Horse, had a very exciting time. He travelled down from Johannesburg in a dirty coal truck, packed in with others like sardines as far as Standerton. When they arrived, their half of the train was left behind, and it was not till after much time and trouble had been wasted, and a tip of £10 given to the guard, that he and three others were allowed to go on in the guard's van of the military Boer train. The burghers who were with them evidently thought, from their knowledge of Dutch and their karkee cycling dress, that they were burghers too, so our friends humoured them, gave them spirits, and got on all right till they reached Sandspruit. Here a young Boer, who knew A—— well, came up to him, and said, 'Look here, you four fellows had better get off quietly as soon as you can. I won't be responsible for my men, for I can't trust them—so get off now.' And off they went, nineteen miles across country to Charlestown, with only a ginger nut each for food all the way! You can imagine how fagged they were before they reached Newcastle, and got a square meal. A—— says that he found the conversation of the Boers in the train very interesting. The older men were all against hostilities, but the young bloods boasted they would 'soon be eating fish in Durban.' They all agreed that they didn't intend to stick by the guns—the guns must take care of themselves; and that as most of the gunners would be Germans, it would be all right. Letters from up-country speak much of the distress of the English who are living in the Free State. The men had all hoped that, if commandeered, they would be despatched only to keep order among the Basutos. But to their horror, they are being sent on active service against their own countrymen, put in the front, and warned that if they shoot in the air, or do not shoot at all, the Boers will fire on them. Is it not horrible?"

One little touching incident before I close. The rector of St. Mary's, Johannesburg, sent his motherless little daughter of three down to Durban to be out of harm's way, and was fearfully troubled at being unable to hear of her arrival. At last, after six weeks of cruel suspense, he learnt that the letter in which her cousins wrote to tell him she was safe in their keeping had also contained little scribbles from his baby for her "dear father," and as these hieroglyphics had been in red pencil, the Johannesburg officials had detained all the letters till they should have succeeded in reading the cypher, which they believed must contain important news!

### The Treatment of the Baby's Cord.

Some correspondence has recently taken place in our columns in regard to a subject of much interest to those engaged in monthly nursing, namely, the treatment of the umbilical cord. The importance of the subject arises from the fact that if the cord is not properly cared for, various accidents, such as tetanus, inflammation of the cord, or even general infection of the whole system, giving rise to pyæmic affections of the liver and other organs, may occur; and its interest is added to by the fact that the text books vary somewhat in the directions which they give as to the manner in which the cord of the new-born child should be dealt with. The older method was the dry one. The cord, wrapped up in a square of toasted and singed linen (*i.e.*, linen rendered aseptic by heat), was laid flat against the abdomen of the child, against which it was held by a binder, and every care was taken to prevent it getting wetted. Of necessity the infant was not placed in a bath until after the separation of the cord. This is an admirable method so long as it can be carried out in its entirety. The cord dries up, shrivels, and when at the end of five or six days the linen is removed, the whole cord is found to have separated and the navel to have healed. But it involves not giving the infant a complete bath, and this is to a certain extent an evil. But what is worse is that while, for the sake of the navel, one is depriving the infant of its bath, the chances are that the navel is all the time getting soaked in a much more objectionable manner; so that, good as is the plan in many cases of not touching the dressing until it is time for it to be removed altogether, it here and there leads to disaster. The cord and its dressings get soaked with urine, or gets wet with perspiration, and instead of drying up it decomposes, and inflammation is set up in the tissues around. The other method is to bathe the infant every day, letting the dressings wash off in the bath, and then to dress the cord afresh each time. It has to be confessed that this plan, while conducing greatly to the cleanliness and sweetness of the child, involves the cord in fresh risks every time. Still, if proper care is taken the risk on the whole is probably less than that of leaving the wrapped up cord to take its chance, especially in district work, where the child has to be left for many hours in unskilled hands. The great thing to bear in mind is that the natural process is for the cord to dry and shrivel, and that the worst thing which we can do is to apply substances which can in any way retain the moisture and keep the cord in a soft and decomposable condition. For this reason all ointments and greasy applications are bad. They form a sort of waterproofing, and by keeping in the vapour prevent the natural evaporation of the part. For the same reason starchy applications are not to be encouraged. So long as they keep dry they do no harm, but when they become wet and then dry up they are apt to produce a more or less waterproof cake which prevents the drying of the parts below. The application of oiled silk or guttapercha tissue is to be absolutely tabooed. A thin layer of absorbent wool, or the old-fashioned singed rag, probably forms the best dressing, but where the daily bath is given and the dressing has to be applied every day, one must remember that the daily bath is a daily-repeated opportunity for the occurrence of septic infection, and that it is a distinct advantage to combine the dressing with the application of some form of antiseptic, or preservative from decomposition, for which purpose nothing is better than a puff of boracic acid. It is a little apt, under the influence of moisture, to run into lumps, and some people prefer to mix it with a little oxide of zinc or with fullers earth, but this is not a matter of great importance. If it should be found that the cord is beginning to become in any way offensive it should be dabbed with carbolic acid lotion (1 in 40) each time it is dressed, but in such case a moderately thick layer of absorbent cotton should afterwards be placed between the cord and the skin of the abdomen so as to protect the skin from the action of the remains of the acid, and only a very thin layer over the cord. If the slightest redness should show itself round the root of the cord a little iodoform may be applied with a camel-hair brush around the root each time it is dressed and after it is dried.

Now in regard to getting what nurses call a flat navel, it is perfectly right to apply a flat pad so as to prevent protrusion, but it must not be imagined that when protrusion occurs it is of necessity the nurse's fault. Generally, the opening in the abdominal wall has nearly closed at birth, in fact, only admits of the passage of the blood vessels, and when the cord drops off there is no opening left. But this is not always the case, and sometimes not only is there a considerable opening in the abdominal walls, but the peritoneal cavity itself extends some little distance into the cord. Practically there is a hernia even from birth, and although in such cases it is possible by great care and the constant use of a flat pad, to prevent this hernia from enlarging, more really seems to depend upon the child being kept happy and comfortable, and free from cough and straining, than upon all the nurse can do with pads. If further protrusion can be prevented, however, these cases may get quite well; so far as its navel is concerned, the child has been born prematurely, that is all, and if the bowel can be kept in place the changes which ought to have occurred before birth will gradually take place, and the ring will close. The difficulty comes when, from the stretching of the navel, caused by straining, a sore is left at the point of separation of the cord, because in these cases it is by no means an easy matter to apply sufficient pressure to keep the rupture in its place without at the same time irritating the ulcer. In such cases it is always best to ask the medical man in attendance to see the navel.

### Appointments.

Penrith Jubilee Cottage Hospital.—Mrs. Louisa M. Stanning has been appointed Nurse-Matron. She was trained at the Royal Infirmary, Liverpool, and Huddersfield Infirmary. Mrs. Stanning has been head nurse at the General Hospital, Newark; head nurse at the Royal Bath Hospital, Harrogate; and matron at the Nicholson McKenzie Memorial Hospital, Strathpeffer, N.B.

### Minor Appointments.

Warwick Workhouse Infirmary.—Miss Annie Bird has been appointed Superintendent Nurse. She was trained at the Royal Southern Hospital, Liverpool, and has subsequently been night staff nurse at Paddington Infirmary, assistant nurse at Woburn Workhouse Infirmary, assistant nurse at Christchurch Workhouse Infirmary, temporary assistant at Plymouth Workhouse Infirmary, and superintendent nurse at Rugby Workhouse Infirmary. She holds the L.O.S. certificate for midwifery.

Infectious Diseases Hospital, Gallows Hill, near Hertford.—Miss Mary Isabelle Libwall Peirce has been appointed Nurse Superintendent. She was trained at the Monsall Fever Hospital, and has also had general training. She has since been on the staff at the Monsall Fever Hospital; sister-in-charge at the Sanatorium, St. Helens; and sister-in-charge at Bagthorpe, Nottingham.

Cresland Moor Workhouse Hospital.—Miss Anne Hadfield has been appointed Superintendent Nurse. She was trained at the Huddersfield Infirmary, and has held the position of children's caretaker at Glossop.

### Everybody's Opinion.

[Correspondence on all subjects is invited, but we cannot in any way be responsible for the opinions expressed by our correspondents. No communication can be entertained if the name and address of the correspondent is not given, as a guarantee of good faith but not necessarily for publication, or unless one side of the paper only is written on.]

### A SUGGESTION FOR NURSES OF A CERTAIN AGE.

"An Inquirer" writes: When so much is written about nurses not getting work when over 40 or 50 years of age, it surprises me that some do not think of going to the outskirts of our large towns and villages to nurse among the artizan class. I have known of two places quite lately which could not get women of any sort to nurse the inhabitants, principally in confinement cases. In both cases the town "Gamp" had died, and no one was left, willing even to help the doctor. There were no funds to support a nurse, but the people were capable of paying small fees, which, if a nurse had a small income of her own, would surely help her to live more comfortably.

### THE BABY'S CORD.

"M. F." writes: I think "Perplexed" will find gauze tissue still more satisfactory than absorbent wool. I dust the cord thoroughly with starch, zinc, and calamine—the latter prevents the powder caking—then, after making a hole in the centre of the gauze, I slip the cord through, turn it up, dust with powder again, place a pad of gauze on the navel, then the flannel binder. I renew the dressings night and morning, but do not put the baby in a bath. If "Perplexed" carries out these simple directions, I do not think she will ever have any trouble with the cord.

### WORKHOUSE NURSES AND THE BATHING OF PATIENTS.

"A Workhouse Nurse" writes: I cannot say how indignant I felt on reading the account of the nurse who has been dismissed by the workhouse master for refusing to bath a male patient. I hope the nurse will report the case to the Local Government Board, who I am sure would not uphold the master. In many workhouses it is the duty of a male officer to bath the chronic and convalescent cases, the nurse blanket-baths the acute and the helpless feeble cases. Failing the provision of a male officer, what objection can there be to a wardsman bathing such patients, providing the nurse prepares and tests the water and remains in hearing of the patient? I have been for years engaged in Poor Law nursing, and I have never had any difficulty with the masters. All have been most kind and considerate in cases of difficulty with male patients. Surely the master in the case referred to must be very inexperienced, and devoid of—well—common sense, to give such orders to the nurse. I hope the nurse who is brave enough to fill the vacancy will have a right understanding with the Board before she takes up her duties.

"A Non-Applicant for the Vacant Post" writes: In your last week's issue you refer to the action taken by a certain workhouse master with regard to the bathing of a male patient by a nurse. It would be interesting to know where he borrowed that delicate idea, or whether it emanated from his own fertile brain. I have not heard of it being the practice anywhere in England, but perhaps the master in question has been on the Continent. Now, no doubt many of the prim and prudish would object to this. But why should they? A nurse has many unpleasant duties to perform, and why should not this be added to the list? Probably the master in question would quite approve of his own wife performing the operation, were it part of her duty to relieve the nurse. And I suppose that if he were in the place of the patient he would also approve. I refrain from expressing my opinion of such a man, as also of a Board of Guardians who could sit down in cool blood and approve of his action. But let me say that, not only the nurses, but the patients themselves, would strike at such a rule. The majority of them have some respect for themselves and the nurses, whether their Guardians and master may have any or not.

### ONE OF THE FAMILY.

"E. J." writes: I have read with much interest the different ideas of nurses how they should be treated in private houses. I have had many years' experience in private nursing, and have received the greatest kindness and consideration from my patients and their friends. I quite agree with "Vera" that nurses make unnecessary trouble for themselves. If a nurse is not received as "one of the family," what possible difference can it make to her if she is a true nurse? Nothing is "unprofessional" which a nurse does for the comfort of her patient or the patient's friends. We must expect little slights sometimes, but it is not worth the unhappiness some nurses seem to think it is. I am quite sure if a nurse does her work cheerfully and conscientiously, she will receive far more kindness and consideration than unkindness.

"One Who Lives to Learn" writes: The correspondence raised, more or less directly, by my question in an August number about "Breakfast in the Bedroom" has been both instructive and diverting to follow. I wish, however, we could manage to keep more to the point, and, above all, that we could avoid all these anonymous digs at one another. It is just as I said; no one can open her mouth without being swooped down upon, sneered at, and lectured, with, or without, religious allusions dragged in. The most inoffensive expressions are twisted into a meaning that was never intended, and much tall talk follows about angels in houses and incessant self-sacrifice—(save the mark!)—with criticism of the "petty" spirit displayed by any nurse who desires to take her necessary nourishment in a civilised and hygienic manner. With regard to what is "unprofessional," it is unprofessional in the sense in which "A Nurse from Worcestershire" used the word, for a sick nurse to "cook the meals and help to keep the house" (note she does not say the patient's room) "in order." She might willingly do it; it might even be, as things were, her plain duty to do it; and this nurse pointedly refrains from saying that she would object if it were so. But it would not be less unprofessional than for the cook to turn to and trim the family hats, or for the housemaid to teach the children their alphabet. One is engaged—one "professes" it—for a definite purpose, and that purpose is to nurse, and in every way care for, the sick. As for counting as a member of the family, it would, I think, be presumption to expect it, and would show great want of experience and knowledge of the world. But when "the family" treats one as "a member" at all the other meals, why should breakfast be singled out to be served in a bedroom at its least-aired time of day? I am glad the question has been threshed out. Experiences have evidently varied very much, and knowledge is useful. But surely it is possible, in other controversies that may arise, for us to try and fall a little less foul of those from whom we differ; to read their words at least twice before we lay about us; to assume that they may be as well-intentioned nurses as we are ourselves; and to think at least three times before we attack them with some of the most sacred utterances we can find anywhere, thus reserving for God the things that are God's, and not applying them to simple questions of humble, though universal importance.

*** This correspondence is now closed.—Ed. *T. H.*

### To Nurses.

In order to increase and vary the interest in the ***Mirror,*** we invite contributions from any of our readers in the form of either an article, a paragraph, or information, and will pay a minimum of 5s. for each contribution. All rejected manuscripts are returned in due course, and all payments for manuscripts used are made at the beginning of each quarter, i.e., January 1st, April 1st, July 1st, and October 1st.

### Presentations.

THE ROYAL HALIFAX INFIRMARY.—RESIGNATION OF THE MATRON.—The Board of Management of the Royal Halifax Infirmary have received with much regret the intimation of Miss Wharton's intention to retire, after twenty-one years' service as matron of the institution, owing to the fact that she has arranged to take over from Mrs. Horton the Dundee Private Nursing Home, at 33, Magdalen Yard Road, Dundee. Miss Wharton received most gratifying testimonials from the Board of Management, and from each of the members of the hon. medical staff. The testimonial from the board was signed by every member of it. We give some extracts :—

"We, the members of the Board of Management of the Royal Halifax Infirmary, beg to say that Miss Wharton has had charge, as matron, of the infirmary during the last twenty-one years. In parting from her, we desire to express our high sense of the services she has rendered to the institution. The years she has passed here have been eventful years, both as regards hospital management generally, and in the history of our own infirmary. Miss Wharton has proved herself capable of meeting every demand made upon her. In the fulfilment of all the duties which the charge of such an institution involves, Miss Wharton has been most efficient. She gave us invaluable help when the infirmary was removed to its new buildings, and the whole organisation of the institution had to be remodelled. Her kindly manner, her tact and good judgment, as well as her practical knowledge and experience, qualify her in an eminent degree for the work she is about to undertake as head of a nursing home. Miss Wharton carries away with her the best wishes of all of us who have worked with her here."

As a further mark of their appreciation of Miss Wharton's work, the members of the board and the hon. medical staff subscribed for and presented to her a silver salver and a purse of gold. The following presentations were also made to her: Framed photograph of the sisters, given by them; a silver tea service and tray from the staff nurses and the probationers; silver tea spoons and tongs from the district nurses attached to the institution; a silver table dish from the female domestic servants; and a silver gong from the male servants.

ROYAL VICTORIA NURSING HOME, ASCOT.—Miss MacDonall having resigned her post at this home, of which she was the first matron, has received a gratifying testimony of the regard in which she is held by the staff in the form of a handsome gold bangle in case, presented in the names of the following: Staff Nurse Miss James, Nurse Steele, Nurse Vine, Nurse Stacey, Nurse Bell, Nurse Newling, Nurse Bashford, and Sarah Wright. This home, opened by the Duchess of Albany in 1898, has already, under Miss MacDonall's care, acquired an established position in the neighbourhood, and fulfilled the most sanguine hopes of its promoters by its usefulness and success.

DORSET COUNTY HOSPITAL.—Miss Hayes, matron of the Dorset County Hospital, has resigned her appointment, to the great regret of the committee, who have warmly appreciated her work. During the five years she has been with them the hospital has been much improved, and many alterations have been made. Miss Hayes, who leaves with best wishes for her future success, has been presented with many valuable gifts from her nurses and numerous friends. She is starting a home for the open-air treatment of phthisis on Marlden Hill, near Paignton, South Devon, combining moorland air with sea breezes.

HAMILTON BRANCH OF THE Q.V.J.N. INSTITUTE.—Miss Philp, late superintendent at Hamilton, has been presented with a handsome gold bracelet on behalf of the ladies of the committee on the occasion of her leaving to be district superintendent at the Central Home in Edinburgh. During the five years Miss Philp has been at Hamilton she has won universal esteem and appreciation of all with whom she has come in contact. Miss Philp takes with her the good wishes of a large circle of friends to her higher sphere of usefulness.

KENDAL DISTRICT NURSING ASSOCIATION.—Miss Oddy, who has just resigned the post of district nurse at Kendal, was last week presented with an illuminated address and a purse of gold by the committee, doctors, and other friends in appreciation of her six years' service. She also received an ormolu clock from Dr. and Mrs. Sturridge, and a silver-plated cream jug, sugar basin, and tongs from a number of grateful patients. Miss Brown, of Burton-on-Trent, has been appointed as her successor.

### Notes and Queries.

**The contents of the Editor's Letter-box have now reached such unwieldy proportions that it has become necessary to establish a hard and fast rule regarding Answers to Correspondents. In future, all questions requiring replies will continue to be answered in this column without any fee. If an answer is required by letter, a fee of half-a-crown must be enclosed with the note containing the enquiry. We are always pleased to help our numerous correspondents to the fullest extent, and we can trust them to sympathise in the overwhelming amount of writing which makes the new rules a necessity.**

**Every communication must be accompanied by the writer's name and address, otherwise it will receive no attention.**

*Book on Midwifery.*

(62) Nurse —— is very anxious to know what book is the best to study for L.O.S.? She has got "Herman's First Lines in Midwifery" and Miss Honnor Morten's "The Midwife's Pocket Book." She is also attending lectures.

See second half of reply to "Invalid Cookery." You should be specially trained for the L.O.S. in practical work as well as theoretical.

*Printed Certificates.*

(63) Will you kindly tell me if I would be allowed to give printed certificates to monthly nurses after I had thoroughly taught them? I am a qualified midwife and nurse (hospital). I have been working in this parish nearly 14 years as district nurse, but I am working now on my own account, though still taking all the poor mothers for the mission. I was thinking of giving free training and certificates to monthly nurses in return for their work among our poor mothers, because the fees (5s.) are too small for me to pay a nurse, and I am always too tired when my work is done to give lectures for the L.O.S. I am well known here, and I shall be so grateful for any advice you can give me.—*St. Alban's.*

You can, of course, give printed certificates; but of what value will they be?

*Splint.*

(64) Can you tell me of something that could be used to soften an outgrowing bone so as to enable it to be straightened into its proper shape again. Of course it would have to be something of a perfectly harmless nature. I should be so much obliged and so thankful if you could do so.—*F. M. S.*

You had better consult a properly qualified medical man.

*Emigrant.*

(65) I am a nurse with two years' training, with experience since then of private work. I am most anxious to go to America or Australia, and would like to take charge of a patient on the voyage. I have advertised, but unsuccessfully. Could you tell me where to apply?—*F. M. H.*

Apply for knowledge of opportunities and prospects of work to the secretary, the Emigrant's Information Office, 31, Broadway, Westminster Sometimes the secretaries of the steamship companies hear of passengers requiring the services of a nurse, but it is a slender chance. Berhaps another advertisement might be more successful.

*Visiting Nurse.*

(66) Would any of your readers who know anything about the scheme of private nurses paying daily visits to their patients instead of living in the house kindly let me know how the plan works? I should be glad to receive a few hints as to fees, times of visiting, &c.—*Enquirer.*

The scheme works very well when well managed in a suitable locality. It is, in fact, district nursing amongst a class of patients who pay remunerative rates for the attendance. The nurse visits the patient once, twice, or oftener if necessary a day, and charges according to the amount of work, the distance away, &c. The fees vary from 2s. 6d. to 5s. a single visit, but this, of course, is ruled by the circumstances of the patient and the case. It is in some cases a good plan to take a fee by the week.

*Systematic Training.*

(67) At the North Staffordshire Infirmary, Stoke-upon-Trent, do the probationers receive a systematic course of theoretical training and lectures in nursing?—*E. N. S.*

Why does our correspondent ask this question? We have no reason whatever to suppose that the system at the North Staffordshire Infirmary is different from that of similar institutions.

*To Lend.*

(68) 1. May I venture to ask if any of your readers have the "Life of Sir A. Clarke" or the "Life of Dr. Harley," which they would lend me upon any terms? 2. Will you kindly say what a private nurse usually takes with her while nursing, syringes and such like?—*A St. Thomas's Nurse.*

1. You would be able to obtain the book from any circulating library in connection with Mudie or Smith for a small fee. 2. A private nurse is not expected to supply her patients with medical equipments.

*Sanitary Inspectors.*

(69) I should be much obliged if you would tell me as much as possible about lady sanitary inspectors, and if it is necessary to be a nurse to become one? Is there much demand for them? At what institute or classes can one get the best training? What is exact nature of their work, length of their working day, &c.? Are there any books on the subject?—*R. B.*

Full particulars may be obtained at the Sanitary Institute, Margaret Street, W. It is not necessary to be a nurse in order to become an inspector. There is not a great demand for them, and their work and hours vary according to the nature of the appointment.

### Travel Notes.

By Our Travelling Correspondent.

### XL.—FLYING SOUTH.

The season has again come round when those who are unable to stand an English winter are thinking of migrating to Southern regions. I cannot flatter myself that those intending travellers will remember my advice of last year, or that they will take the trouble to look it up. The shortest way will be to give it you again.

### Time to Leave England.

This is almost always delayed too long. Naturally, those who only seek warmer climes for pleasure and comfort, and are not compelled to do so from motives of self-preservation, are sorry to lose the agreeable house parties of an English autumn; but those whose health imperatively demands absence from our changeable weather should not delay their flight beyond the end of October. The terrible yellow and black fogs which we enjoyed a fortnight ago gave warning that those delicate on their chests should be up and away. Leave England at the end of October, and do not return until the end of May. All the good achieved by a winter in the South is neutralized, if not positively quenched, by returning to England to encounter cruel north-east winds, and a month makes but little difference; if you are compelled to absent yourself from home for six months, you may just as well make it seven and lessen the danger of meeting with a bronchial attack on your return.

### The Relative Merits of Apartments and Hotels.

If money is no object (but, alas, it is to most of us) I think the ideal thing is a suite of apartments in an hotel. You are thus saved all trouble of housekeeping, servants, &c. When economy has to be considered I fancy it is still a question whether you will gain much by living in rooms of your own. In most health resorts lodgings are dear, and you are subject to continual unexpected expenses which soon run up the total to almost the same as the cost of living in a reasonable hotel. Moreover, to live comfortably, and at the same time economically, in your own rooms, it is essential that you not only understand the language of the country, but are not above going into the market with your femme de menage and getting in supplies. As a broad rule I should say that apartments are a mistake for less than four months, and unless you understand the language and ways of the people amongst whom you are temporarily located. Apartments are generally but meagrely provided with what we consider the essentials of comfort. Bed and table linen is usually abundant, but such homely, useful friends as kettles, jugs, trays, dusters, &c., are conspicuous by their absence, and these simple necessaries are dear to buy; you ask for a tin kettle, and you are invited to inspect a vast iron receptacle that even empty is no light weight; cost from 4 to 7 frs. Our light, cheap little treasures are quite unknown to Italians and French. Basins, too, are few and far between, and when small meals are required for an invalid it is very inconvenient; and as for trays, they are luxuries not generally met with. Still, with all these drawbacks, living in apartments for any length of time is on the whole cheaper than hotel life, but is the game worth the candle? that is the question. You save some money certainly, but you lose considerable comfort.

### Clothes Required for a Winter Abroad.

It is a mistake to suppose that the Riviera or southern France and Spain are always warm, whilst Florence and Rome are distinctly cold, but the great difference lies in the almost continual presence of the sun; whereas in England, though perhaps not particularly cold, there is during the winter so little sun and often so very much mist and fog that a delicate person is almost entirely confined to the house, which, I need hardly remind you, is the worst possible thing for them. The object gained by wintering abroad is that as much oxygen and sun may be absorbed into the system as possible. To this end be out of doors continually, but suitably dressed. Let all your clothes be woollen, but of different thicknesses to suit different states of the temperature. As spring advances and the sun gains great power there is no reason why cotton skirts should not be worn, always provided that warm underclothes are never laid aside, such as woollen combinations high to the neck, knickerbockers, and a woollen petticoat as well for windy days. It is a good plan to have silk blouses lined with thin flannel; they always look smart, and are more convenient than cotton ones with under bodices. Take a light wrap over your arm when out walking, because the sudden chill experienced when turning out of the blazing sun to a shady side street is very dangerous. For real comfort I think nothing equals tailor-made coats and skirts. By a judicious plan of having one thick and one thin you can be always suitably and well dressed. For evening wear simple silk frocks and blouses are the best; it is not good form to be very much dressed at *table d'hôte*. With regard to shoes and boots, you will not often encounter mud as we do here, so that it is unnecessary to have very thick boots. One pair moderately substantial and two pairs of Oxford shoes ought to see you through the winter. English-made feet coverings are extremely dear abroad, so take what you want with you. Always wear woollen or silk stockings, cotton and lisle thread are not advisable. A comfortable tea-gown is a luxury that one fully appreciates abroad; the change is so delightful after a long, hot walk or drive; let it be of merino, crêpon, or silk. A thoroughly warm dressing gown and a good shawl and railway rug are almost necessities.

### Essential Comforts not to be Forgotten.

Amongst these I place a tea basket. Tea is dear and badly made on the Continent. Always make it in your own room with a spirit lamp. It is unnecessary to buy one of the expensive articles to be seen in good shops. You can arrange everything for about 5s. in a champagne basket. I gave a receipt for its construction last winter. Take with you an indiarubber hot water bottle, several towels (foreign ones are small), a couple of 6d. Japanese trays, and a folding drinking cup. It is wise to take a shaving glass, even if you are of the feminine gender; they are made in three folds, and hang anywhere by a chain; the looking glasses are generally bad, and so hung that one can see absolutely nothing, besides being often of a quality that gives one a pervading green tinge and a general tendency in every feature to run sideways.

---

### TRAVEL NOTES AND QUERIES.

Oberammergau (Stevenage).—I will keep your envelope until all the printed lists are out, which will probably be in another month. Dr. Lunn's tours at £25 4s. will probably be very comfortable, and not quite so crowded as Cook's or Gaze's. The latter firms have not yet issued their full particulars. No; I do not think the sum will cover all expenses, because there are certain small disbursements which are not counted in that sum, and yet which are unavoidable, such, for instance, as the one mark charged extra on every seat on the German railways. With Lunn's tours this is paid for you, and included in the 24 guineas going out, but as you will return without the conductor you must defray it yourself; it will come to a few shillings only, the sum depending on the route taken. Another expense is that gratuities to hotel servants are not reckoned in the 24 guineas. I am afraid you cannot reckon this at less than £2 for twenty days, but this sum will cover everything. I should myself consider £2 10s. will cover all extras. But you should take more with you. It is quite unnecessary to spend it, but it is not safe to travel without at least £5 to spare. If you do not wish to spend so much why not come home immediately after the play, making only fifteen days instead of twenty. Watch for a further answer. A little later I shall personally see the various tourist agents and learn all arrangements. The accommodation is limited, and therefore there is unavoidable crowding, but I think five ladies in one room is exaggerated; but it is quite true that lodging at Oberammergau is very rough and ready, but it is only one night you see, and the kind of people you will meet will not be objectionable.

